# Vaginal Bleeding as Initial Presentation of an Aggressive Renal Cell Carcinoma: A Case Report and Review of the Literature

**DOI:** 10.1155/2018/2109279

**Published:** 2018-06-04

**Authors:** Antonio R. Jimenez, Maria del Mar Rivera Rolon, Eduardo Eyzaguirre, Cecilia Clement

**Affiliations:** Department of Pathology, University of Texas Medical Branch, 301 University Boulevard, Galveston, TX 77550, USA

## Abstract

**Introduction:**

Renal cell carcinoma is the third most common urogenital cancer. In some patients, it can metastasize to distant organs. Metastasis to the vagina is extremely rare.

**Case Presentation:**

A 54-year-old female with unremarkable history presented to the clinic with a chief complaint of vaginal bleeding. Further examination identified a pedunculated mass on the vaginal wall. Histologic examination revealed a metastatic clear cell renal cell carcinoma. Radiological studies then revealed a left renal mass and bilateral adrenal masses. The patient underwent a nephrectomy, adrenalectomy, and resection of the vaginal mass. The mass in the vagina has since recurred.

**Conclusion:**

We report the first known case of vaginal metastasis as initial presentation of a renal cell carcinoma with rhabdoid features. Postmenopausal women with renal cell carcinoma who present with vaginal bleeding should undergo a thorough inspection of the vaginal wall for the potential of metastatic neoplasms.

## 1. Introduction

Renal cell carcinoma (RCC) is a malignant neoplasm that originates from the renal tubular epithelium. Approximately 60 to 80% of renal cancers are discovered incidentally on imaging studies [1, 2]. The classic presentation of RCC is flank pain, hematuria, and a palpable abdominal mass. However, this presentation is uncommon and occurs in less than 10% of affected individuals [1]. Literature has identified that approximately 30% of patients with RCC present with metastases at the time of their initial diagnosis. Metastasis of clear cell RCC (CCRCC) to the vagina is extremely rare, with less than 100 reported cases in medical literature.

## 2. Case Presentation

A 54-year-old, obese, Caucasian female, a current smoker, presented to the clinic with complaints of intermittent vaginal bleeding for approximately one week and that she “felt something” in her vaginal area. Her past medical history includes diabetes mellitus, hypertension, and an abdominal hysterectomy 30 years ago due to heavy menstrual bleeding of benign etiology. Upon examination, a pedunculated mass was found on the vaginal wall at approximately 7 o'clock. The mass, measuring 2.0 × 1.5 × 1.3 cm, was subsequently excised.

**Figure 1:**
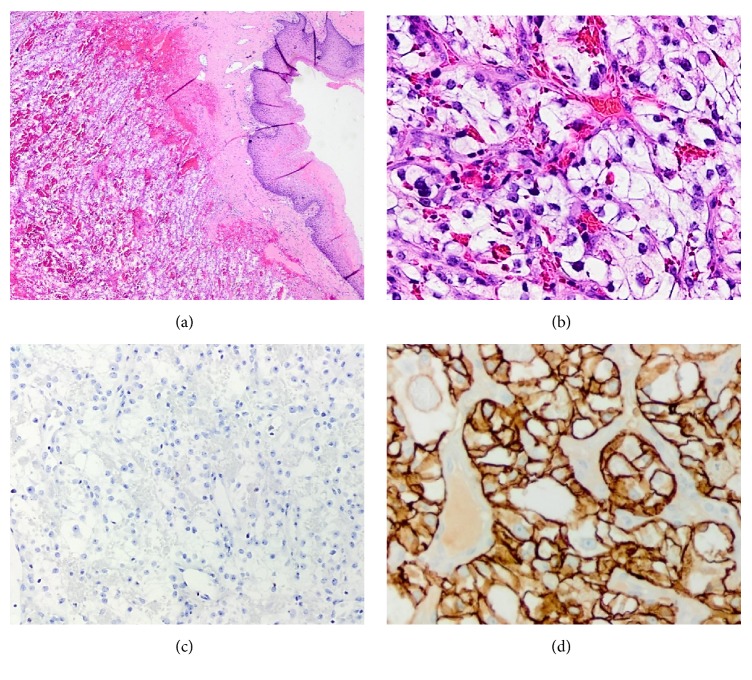
Histology of initial vaginal mass. (a) Vaginal nodule showing neoplastic cells with abundant clear cytoplasm and characteristic delicate vascular network (H&E, ×20). (b) High power view of the vaginal lesion revealed a clear cell carcinoma (H&E, ×200). (c) Neoplastic cells were negative for CK7 (×100). (d) Tumor cells showing complete membranous immunopositivity for CA-IX (×200).

Histologic examination revealed a clear cell carcinoma. Immunohistochemical stains were positive for CD10, PAX-8, and carbonic anhydrase 9/IX (CA-IX) and negative for CK7. A diagnosis of metastatic CCRCC was made (Figure 1). Radiological studies then revealed a left renal mass, bilateral adrenal masses, and enlarged retroperitoneal lymph nodes. A subsequent left radical nephrectomy and adrenalectomy was performed. On gross examination, a unifocal tumor that measured 14.7 cm in its greatest dimension was identified. Pathologic examination confirmed the renal origin of the vaginal carcinoma. The nephrectomy diagnosis was established as CCRCC with rhabdoid differentiation and multinucleated giant tumor cells. The World Health Organization (WHO)/International Society of Urologic Pathologists (ISUP) grade was 4. The tumor invaded into the perinephric fat tissue, renal sinus, and major branches of the renal vein. Tumor necrosis and lymphovascular invasion were identified. All margins, including Gerota's fascia, ureteral and vascular, were free of malignancy. The left adrenal gland was involved with metastatic CCRCC. The final pathological stage was pT3a pNx pM1.

**Figure 2:**
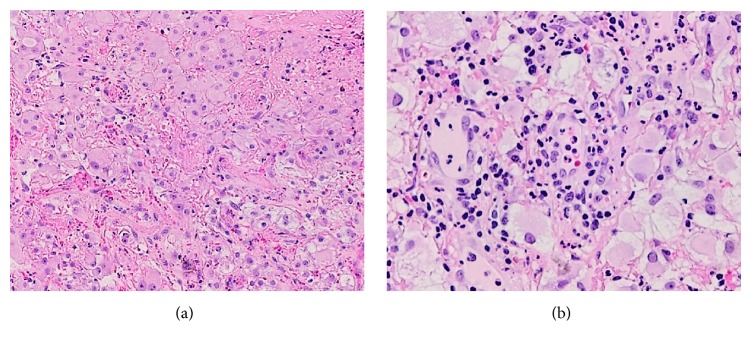
Histology of recurrent vaginal mass. (a) The clear cell renal cell carcinoma showing areas of inflammation and extensive rhabdoid differentiation (H&E, ×100). (b) High power view of the rhabdoid cells observed in the recurrent vaginal excision (H&E, ×200).

Multiple pulmonary nodules, measuring up to 4 mm, and a right adrenal nodule were observed on imaging. The patient is now receiving adjuvant targeted therapy (Sunitinib 50 mg). The lung nodules are no longer seen, and the right adrenal nodule had decreased more than 30% in size. Interestingly, her vaginal mass recurred, increased in size, and changed in shape since her last resection. She, thus, underwent a partial excision of the new lesion on the vaginal wall. The pathologic examination revealed a CCRCC, with extensive rhabdoid differentiation this time (Figure 2).

## 3. Discussion

Renal cell carcinoma has the potential to metastasize in roughly 30% of cases [3, 4]. Metastatic RCC can hematogenously disseminate into the lung, bone, adrenal glands, liver, lymphatic nodes, and brain. In addition, vaginal wall metastasis is also possible; however it is a rare event. The first dated case of RCC-associated vaginal metastasis was described by Penham in 1906. To date, medical literature has reported less than 100 cases of secondary vaginal tumors from RCC. The great majority of metastatic CCRCC to the vagina present as recurrences, after the diagnosis of the renal tumor has been established. It is rare for CRCC to present clinically as a primary vaginal tumor with postmenopausal bleeding. The histologic differences between primary vaginal clear cell carcinomas (CCC) and metastatic CCRCC vaginal lesions are subtle and differentiation in a small biopsy can be challenging. Typically vaginal CCC show variable morphologic patterns including solid, tubulocystic, and papillary, with presence of hobnail cells. CCRCC characteristically shows alveolar, acinar, and nested patterns and papillary architecture is not a feature of CCRCC; in addition, a prominent network of branching small, thin-walled blood vessels is characteristic and diagnostically helpful. Therefore, due to the presence of extensive overlapping morphologic features between both tumors [5], it is recommended to have the kidneys evaluated in cases of vaginal neoplasms with clear cell features. This examination would thus prevent a misdiagnosis of a primary vaginal adenocarcinoma. Immunohistochemistry is helpful in differentiating these entities as CA-IX and CD10 are most frequently expressed in CCRCC than Mullerian CCC, whereas CK7, Napsin-A, and methylacyl-coenzyme-A racemase (AMACR) show a reverse pattern of expression [3]. Although PAX8 is a very important marker for the diagnosis of metastatic RCC, it is also expressed in CCC of Mullerian origin and therefore is of no use in distinguishing a renal versus a vaginal primary [6].

In most recorded cases of vaginal metastases, a firm, solitary lesion was found on the lower third of the anterior vaginal wall [7]. In addition, tumor-associated vaginal bleeding was the initial presentation of a RCC in most clinical cases. Metastases to the vagina are thought to originate from the retrograde flow of the left renal vein to the ovarian vein and uterovaginal plexus [8]. Tumor emboli then dissipate to the vagina via venous reflux of the ovarian vein. The proposed mechanism may then explain the increased prevalence of vaginal metastases in tumors of the left kidney, as observed in the present patient [9]. Right-sided RCC also have the potential to cause vaginal metastases in patients. It is likewise theorized that retrograde flow between the right renal vein and right ovarian vein is the cause of metastatic dissemination to the vagina.

We present the first known case of vaginal bleeding as the initial presentation of an aggressive CCRCC with rhabdoid features; therefore, the tumor was deemed WHO/ISUP grade 4. There is a 4.7% incidence of the rhabdoid phenotype in CCRCC. These tumors are often associated with an increased stage and an aggressive clinical course [10]. However, the five-year prognoses for non-rhabdoid RCC that have metastasized to the vagina are also poor. The median lifespan of a patient with RCC-associated vaginal metastasis is approximately 19 months [8]. To date, surgical nephrectomies, and adrenalectomies, dependent on the aggressiveness of the cancer, and adjuvant chemotherapy are common utilized approaches for RCC treatment. The removal of the solitary vaginal lesion has also demonstrated favorable prognostic results in some patients. In this patient targeted therapy with Sunitinib was started, and she has completed her 5th cycle. However, during her last visit she stated that the vaginal mass appeared to have increased in size. The reason for this failure in targeted therapies such as the use of Sunitinib in CCRCC may be due to the often multiple independent inactivating mutations in different clonal populations within a single tumor and its metastases [11].

## 4. Conclusion

We describe the first known patient with metastasis to the vagina in a CCRCC with rhabdoid differentiation. The most notable clinical presentation in the case was vaginal bleeding. The incidence of RCC metastases to the vagina is extremely rare; however, it should be considered as a potential differential diagnosis in patients who present with vaginal bleeding or mass.

## Data Availability

Our conclusions arise from the evaluation of the histopathologic findings described in this study. No other data can be released due to patient confidentiality.
